# Supplementary material for: The Systems Biology Research Tool: evolvable open-source software
Source: BMC Syst Biol. 2008 Jun 29;2:55. doi: 10.1186/1752-0509-2-55 (PMC2446383; doi:10.1186/1752-0509-2-55)
Supplement: Additional file 1 — SBRT Archive. An archive of the current version of the Systems Biology Research Tool. [file 1752-0509-2-55-S1.zip › sbrt-1.4.0/doc/developers_guide/api/sbrt/kernel/fba/IrrevRxn.html]

IrrevRxn


|  |  |  |
| --- | --- | --- |
| **Overview** \| **Package** \| **Class** \| **Use** \| **Tree** \| **Deprecated** \| **Index** \| **Help** | |  |
| **PREV CLASS**   **NEXT CLASS** | **FRAMES**    **NO FRAMES**     **All Classes** |  |
| SUMMARY: NESTED \| FIELD \| CONSTR \| METHOD | DETAIL: FIELD \| CONSTR \| METHOD |  |


---


## sbrt.kernel.fba Class IrrevRxn

```
java.lang.Object
  sbrt.kernel.fba.AbstractRxn
      sbrt.kernel.fba.IrrevRxn
```

**All Implemented Interfaces:**: ChemRxn

---

``` public class IrrevRxn extends AbstractRxn ```

This class is used to represent irreversible chemical reactions. Reactions
that proceed nearly to completion may be approximated as irreversible.
Reversible reactions can also sometimes be treated as two irreversible
reactions, with one as the "forward" reaction and one as the "reverse"
reaction.

Instances of this class are immutable.

**Author:**
:   This class was written and documented by Jeremiah Wright while in the
    Wagner Lab.

---

**Constructor Summary**

`IrrevRxn(LinearComb<java.lang.String> reactants,
LinearComb<java.lang.String> products)`
  
          Constructs a new irreversible reaction from the provided reactants and
products.


| **Method Summary** | |
| --- | --- |
| `boolean` | `isEquivalent(ChemRxn rxn)`              Indicates if the provided chemical reaction is stoichiometrically equivalent to this reaction. |
| `boolean` | `isReverse(ChemRxn rxn)`              Indicates if the provided chemical reaction is the reverse of this reaction. |
| `java.lang.String` | `toString()`             Returns an appropriate string representation of this irreversible reaction. |

**Methods inherited from class sbrt.kernel.fba.AbstractRxn**

`equals, getProducts, getRatio, getReactants, hashCode, main`

**Methods inherited from class java.lang.Object**

`clone, finalize, getClass, notify, notifyAll, wait, wait, wait`

**Constructor Detail**

### IrrevRxn

```
public IrrevRxn(LinearComb<java.lang.String> reactants,
                LinearComb<java.lang.String> products)
```

:   Constructs a new irreversible reaction from the provided reactants and
    products.

    **Parameters:**: `reactants` - the linear combination of chemical species representing the "left-hand side" of the reaction.: `products` - the linear combination of chemical species representing the "right-hand side" of the reaction. **Throws:**: `java.lang.NullPointerException` - if the reactants or products are `null`, or if any chemical species within them is `null`.: `java.lang.IllegalArgumentException` - if • the reactants or products contain a common chemical species, • either linear combination contains a non-zero constant, • a stoichiometric coefficient is NaN, infinite, or ≤ 0.


**Method Detail**

### isEquivalent

```
public boolean isEquivalent(ChemRxn rxn)
```

:   Indicates if the provided chemical reaction is stoichiometrically
    equivalent to this reaction.

    :   **Parameters:**: `rxn` - the reaction to test for equivalence. **Returns:**: true if both reactions are equivalent; false otherwise.

---


### isReverse

```
public boolean isReverse(ChemRxn rxn)
```

:   Indicates if the provided chemical reaction is the reverse of this
    reaction.

    For the provided reaction to be the reverse of this reaction,
    getReactants().equals(rxn.getProducts()) and
    getProducts().equals(rxn.getReactants()) must both return true. The
    provided reaction must also be an IrreversibleRxn.

    NOTE: The way reverse reactions are defined here is very restrictive. By
    this definition, the reactions (2) H_2_ + O_2_
    → (2) H_2_O and H_2_O → H_2_ +
    (½) O_2_ are not the reverse of each other, because their
    stoichiometric coefficients are different.

    :   **Parameters:**: `rxn` - the reaction to test. **Returns:**: true if the provided reaction is the reverse of this reaction; false otherwise.

---


### toString

```
public java.lang.String toString()
```

:   Returns an appropriate string representation of this irreversible
    reaction.

    :   **Specified by:**: `toString` in interface `ChemRxn` **Specified by:**: `toString` in class `AbstractRxn`
    :   **Returns:**: an appropriate string representation of this irreversible reaction.


---


|  |  |  |
| --- | --- | --- |
| **Overview** \| **Package** \| **Class** \| **Use** \| **Tree** \| **Deprecated** \| **Index** \| **Help** | |  |
| **PREV CLASS**   **NEXT CLASS** | **FRAMES**    **NO FRAMES**     **All Classes** |  |
| SUMMARY: NESTED \| FIELD \| CONSTR \| METHOD | DETAIL: FIELD \| CONSTR \| METHOD |  |


---
